# Supplementary material for: Large-scale capture of hidden fluorescent labels for training generalizable markerless motion capture models
Source: Nat Commun. 2023 Sep 26;14:5866. doi: 10.1038/s41467-023-41565-3 (PMC10522643; doi:10.1038/s41467-023-41565-3)
Supplement: Supplementary file 1 — Supplementary Information [file 41467_2023_41565_MOESM1_ESM.pdf]

Supplementary Information

Large-scale capture of hidden fluorescent labels for training generalizable markerless motion capture models

Daniel J. Butler[1], Alexander P. Keim[1], Shantanu Ray[1], Eiman Azim[1*]

[1]Molecular Neurobiology Laboratory, Salk Institute for Biological Studies, 10010 N. Torrey Pines Road, La Jolla, CA 92037, USA.

[*]Corresponding author (eazim@salk.edu)

Number of Supplementary Figures: 9

Number of Supplementary Tables: 1

Number of Supplementary Movies: 6

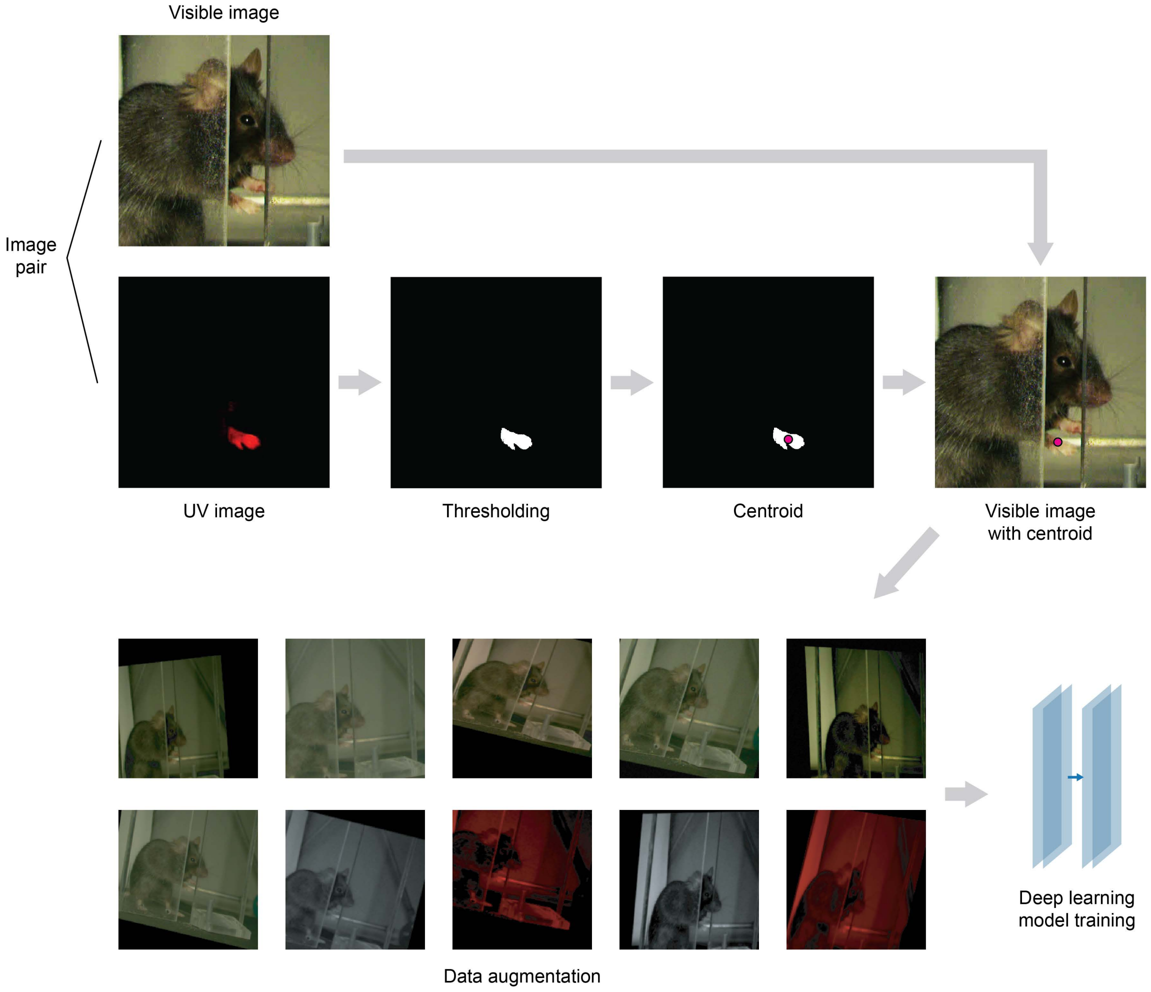

Supp. Fig. 1. Conversion of raw imagery into labeled training data. The data processing pipeline for a visible image (top left) and its corresponding UV image. The UV image is first thresholded to produce a dye mask, and the centroid of the dye mask is computed. The centroid is used as a proxy label for the subsequent visible image. During training, each labeled training image is augmented with scaling, rotating, shear, Gaussian noise, and random scaling of hue. In addition, the image is converted to grayscale or the non-red channels are set to zero, both with a fixed probability, to simulate monochrome imagery and red illumination, respectively (see Methods). Example augmented images are shown.

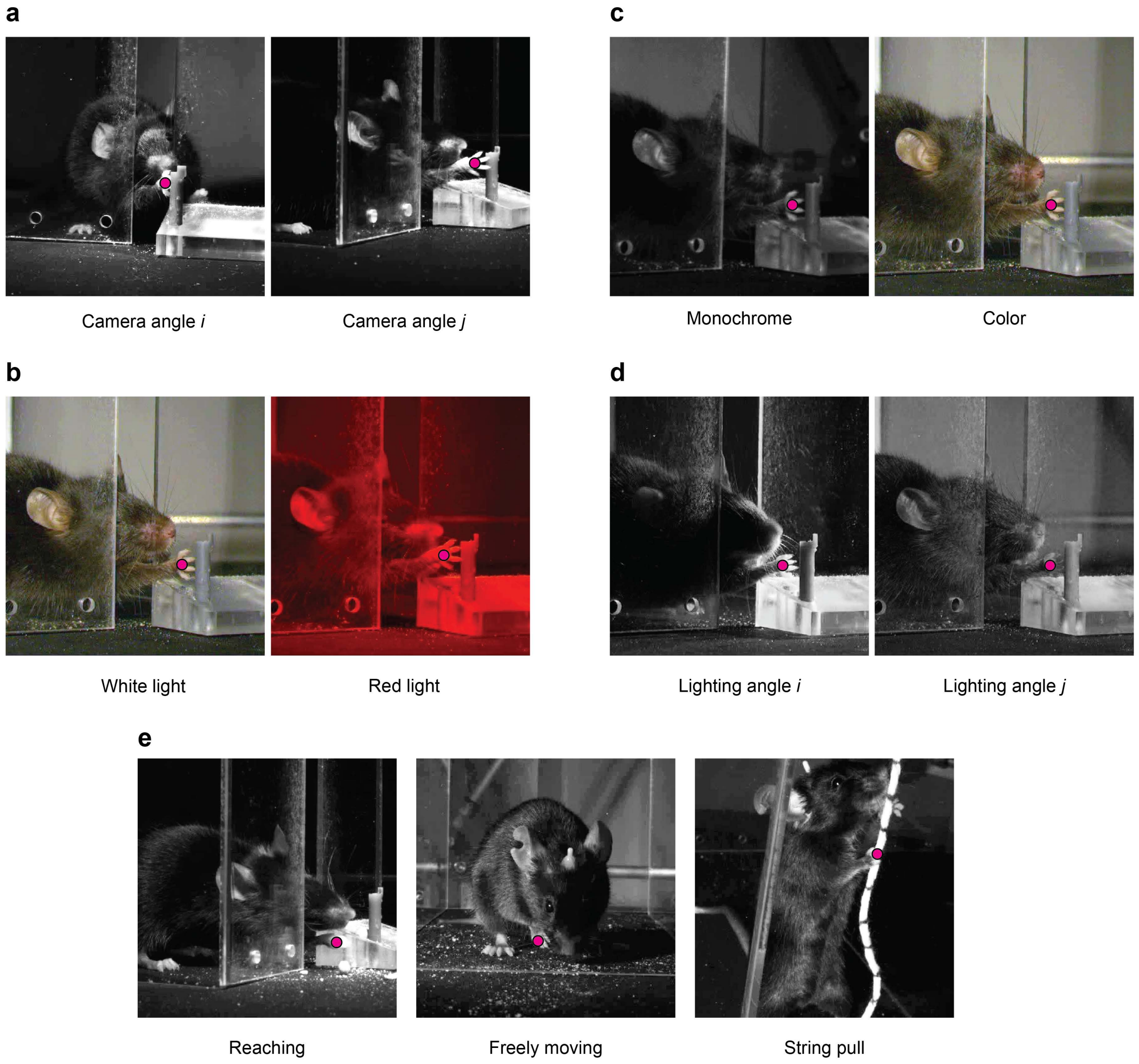

Supp. Fig. 2. Image diversity in the fluorescence-derived mouse dataset. Images captured: **a)** simultaneously from two cameras; **b)** sequentially under white and red illumination; **c)** simultaneously from monochrome and color cameras; **d)** under different lighting angles; and **e)** during reaching behavior (left), freely moving behavior (middle), and string pull behavior (right). Landmark labels (magenta dots) are derived from the corresponding fluorescence images (not shown). Panels **a** and **b** are reproduced from **Fig. 3**.

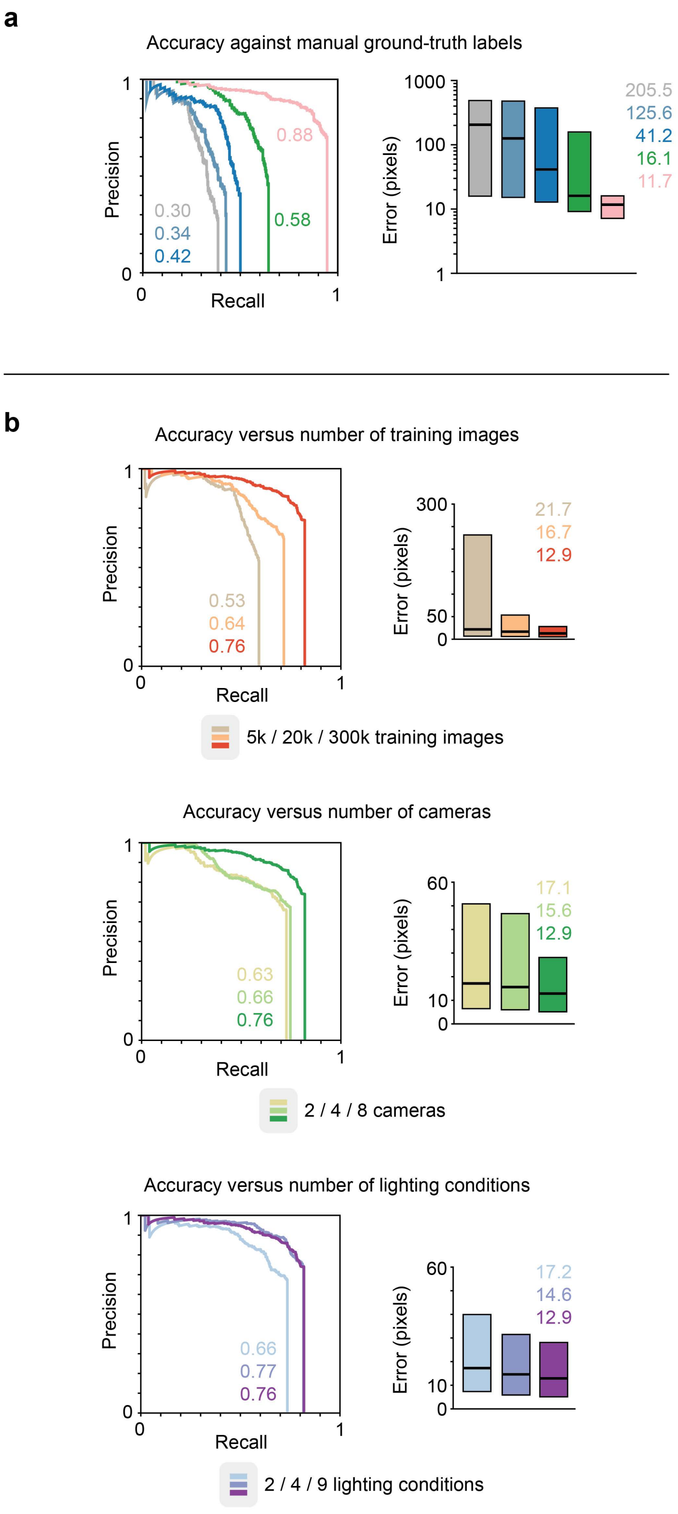

Supp. Fig. 3. Contributions of ground truth source and training set parameters to neural network performance. Precision-recall curves (left; numbers indicate area under the curve) and pixel error quartile plots (right; center lines and numbers indicate median, boxes indicate 25th and 75th percentiles). **a)** Results on the diverse test set, but with manual rather than fluorescence-derived ground truth labels. Values are similar to those produced when evaluating against fluorescence-derived labels (compare to **Fig. 3h**), confirming that fluorescence-derived labels are a valid source of ground truth (n = 600 test images). **b)** Results on the challenge data set for neural networks trained on different subsets of the fluorescence-derived training set. The number of training images, number of cameras, and number of lighting conditions all contribute to area under the curve and median pixel error (n = 4438 test images). Source data are provided as a Source Data file.

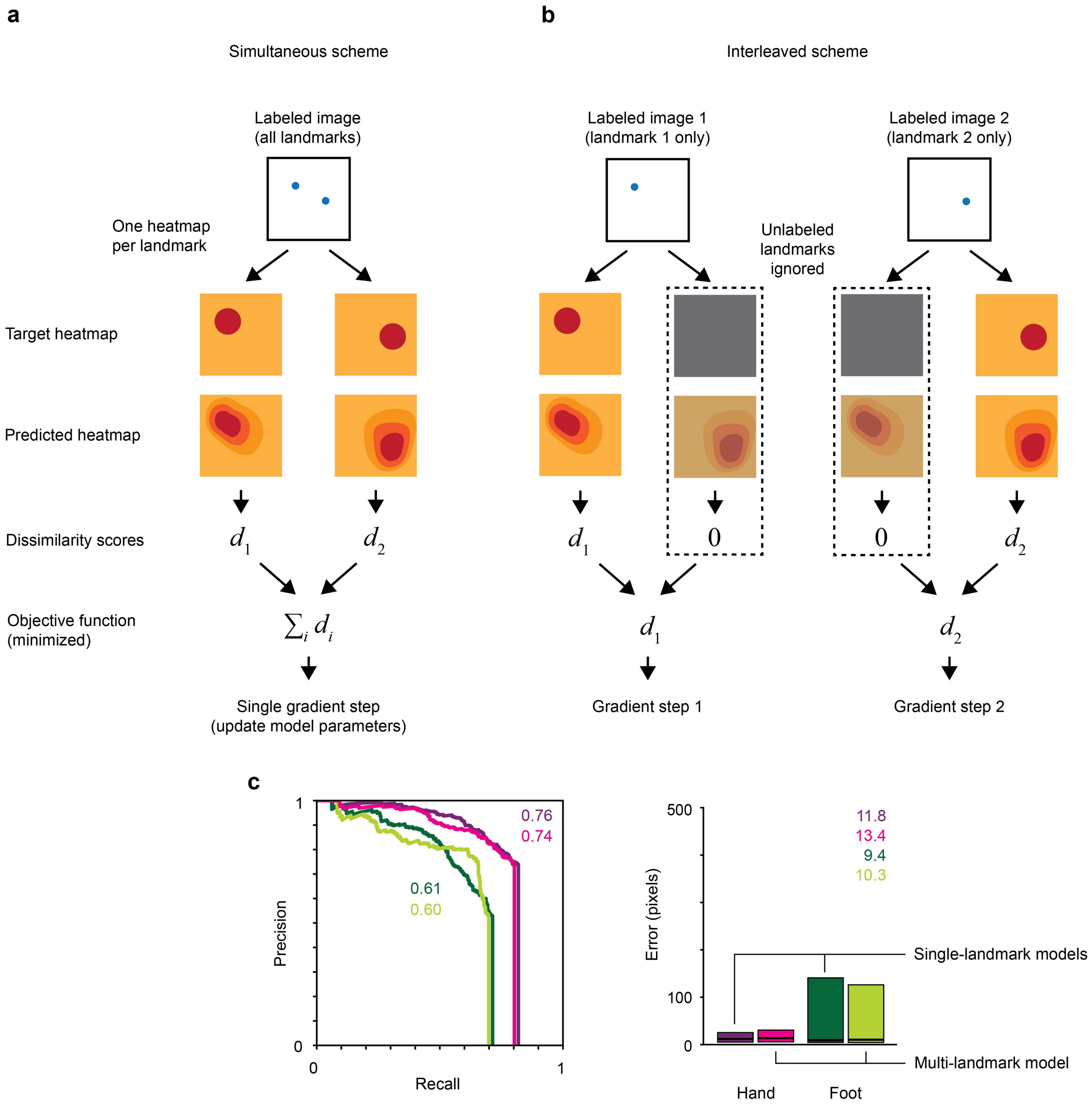

Supp. Fig. 4. Training a multi-landmark model via serial labeling. a) The typical scheme for training a neural network model to predict multiple landmarks. Each training image has labels for all landmarks, and all landmarks contribute to the loss function. **b)** The interleaved scheme used to concatenate multiple datasets with different landmarks labeled. Each image has only one landmark label, and only that label contributes to the loss function. **c)** Precision-recall curves (left; numbers indicate area under the curve) and pixel error quartile plots (right; center lines and numbers indicate median, boxes indicate 25th and 75th percentiles). Panels show results of training a multi-landmark model on two landmarks (right hand in magenta, right foot in light green) using the interleaved scheme and evaluated using the challenge test set (n = 612 test images for hand, n = 288 test images for foot). For comparison, results are also shown for training two separate single-landmark models (right hand in dark purple, right foot in dark green). The multi-landmark model exhibits essentially no loss of accuracy when compared to single-landmark models. Worse performance on the foot versus the hand for both multi-landmark and single-landmark models is likely due to the fact that challenge test set videos tended to prioritize visibility of forelimb over hindlimb, and videos that had both hand and foot clearly visible were lower resolution. Source data are provided as a Source Data file.

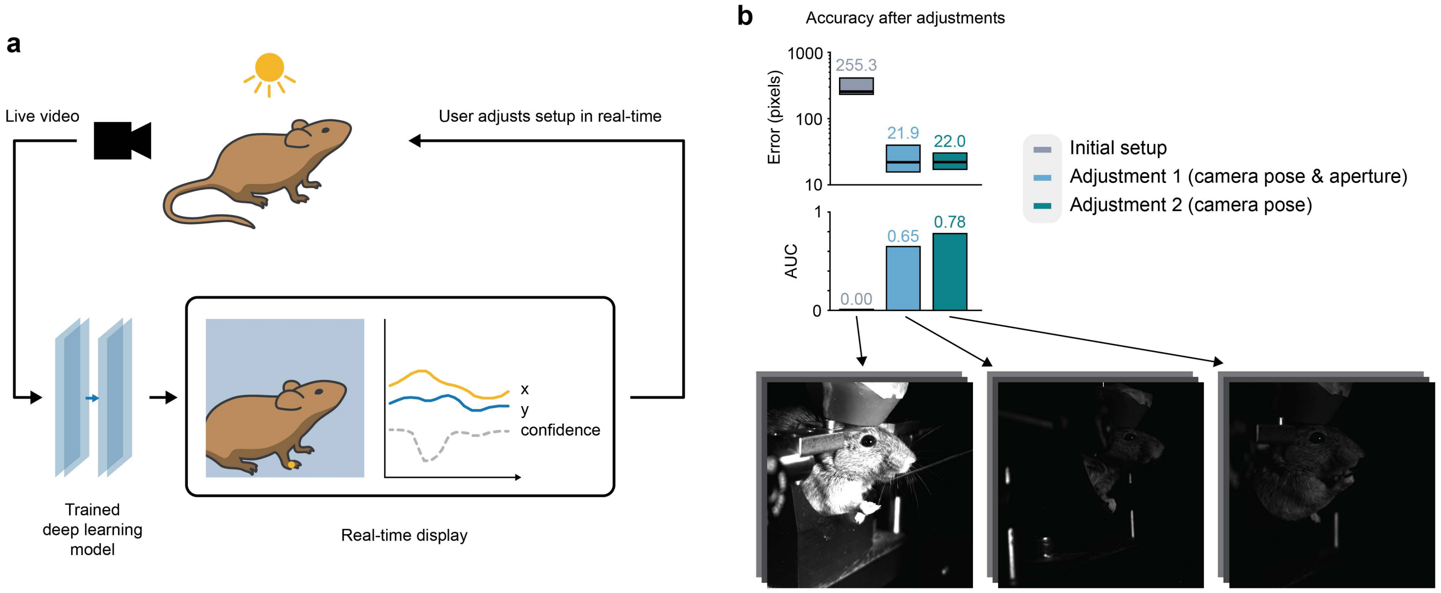

Supp. Fig. 5. Real-time visualization enables interactive adjustment and improved performance. **a)** A live video stream is captured and processed by a trained deep learning model that predicts landmark position. The live video is displayed to the user with the predicted landmark label overlaid and a plot of its most recent 100 x positions, y positions, and confidence values over time. The plot is continuously updated in real-time, allowing the user to adjust the position of the animal, camera, and lighting interactively while visualizing the impact on the model's predictions. **b)** Pixel error quartiles (top; center lines and numbers indicate median, boxes indicate 25th and 75th percentiles) and area under the precision-recall curve (middle) for three different positions of the camera and settings of the camera lens aperture. Sample images from each setup are shown at bottom. Performance increases over time as the user makes adjustments to the setup to produce images that are better suited to the trained model (n = 200 test images for each condition). Source data are provided as a Source Data file.

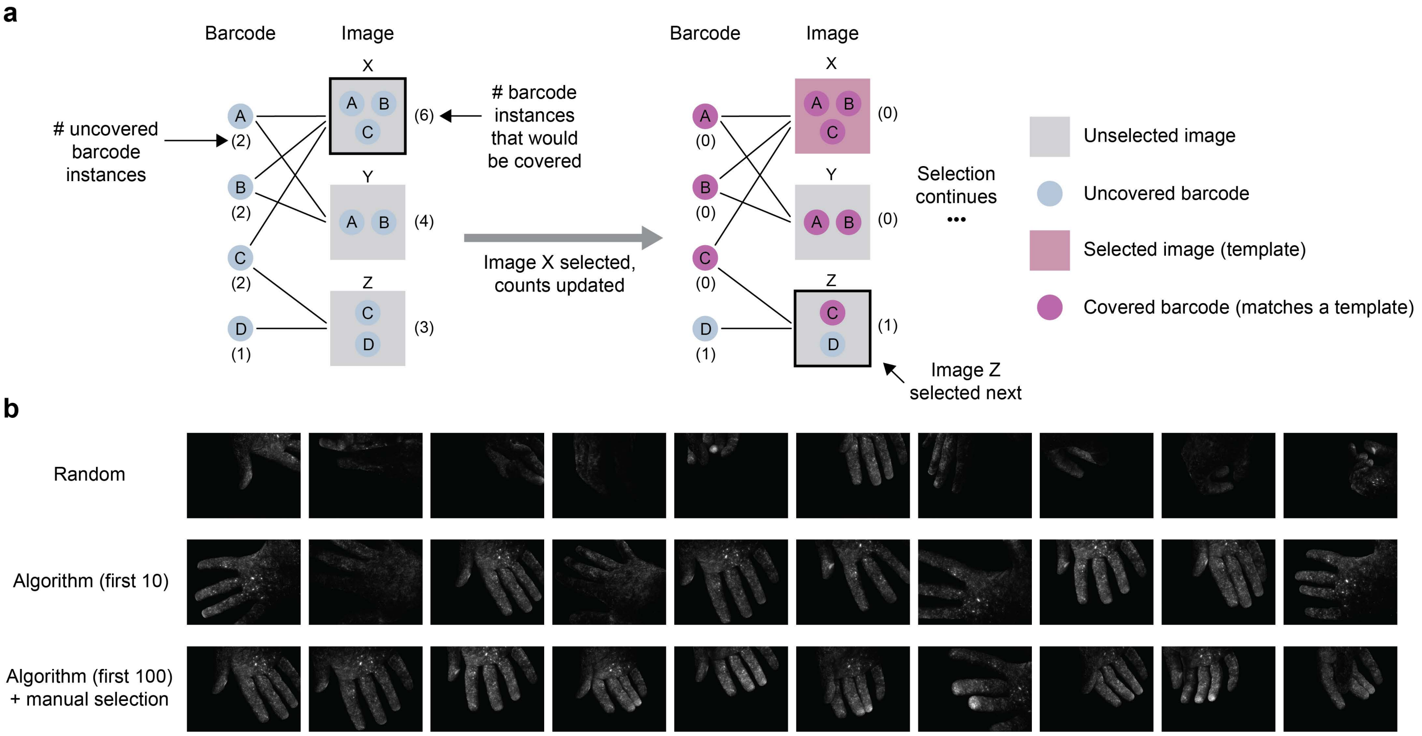

Supp. Fig. 6. Greedy algorithm for template image selection. a) An estimate of the number of barcode instances that would be matched (covered) by each candidate template image is calculated (left). The image with the highest instance coverage (black outline) is selected as the next template image, the counts are updated (right), and the procedure is repeated until the desired number of template images have been selected. **b)** Examples of 10 template images selected randomly (top) and by the greedy algorithm (middle). To train a network that labels landmarks on the palm side of the hand, images that depict that specific region (bottom) were manually selected from among the top 100 images as ranked by the greedy algorithm.

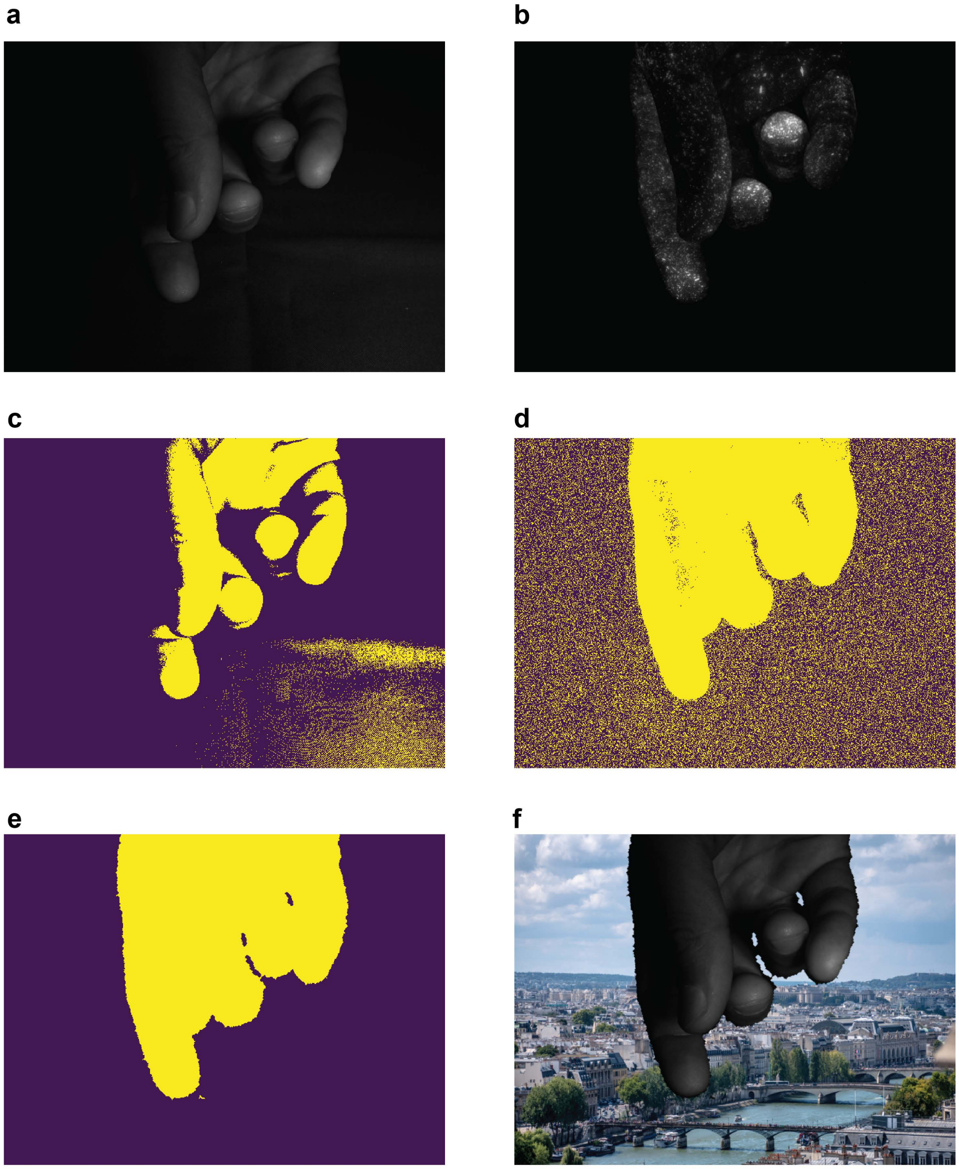

Supp. Fig. 7. Background segmentation and augmentation using fluorescence. a) Visible image; **b)** UV image; **c)** thresholded visible image, which does not provide a clean foreground mask due to shadows; **d)** thresholded UV image, which provides a cleaner foreground mask; **e)** thresholded UV image with morphological erosion to reduce spurious background signal (see Methods); **f)** target object segmented and superimposed onto a synthetic background image, using image from panel **e** as mask. During training, the image background is augmented to increase the model's robustness to any changes in the scene that happen away from the target object.

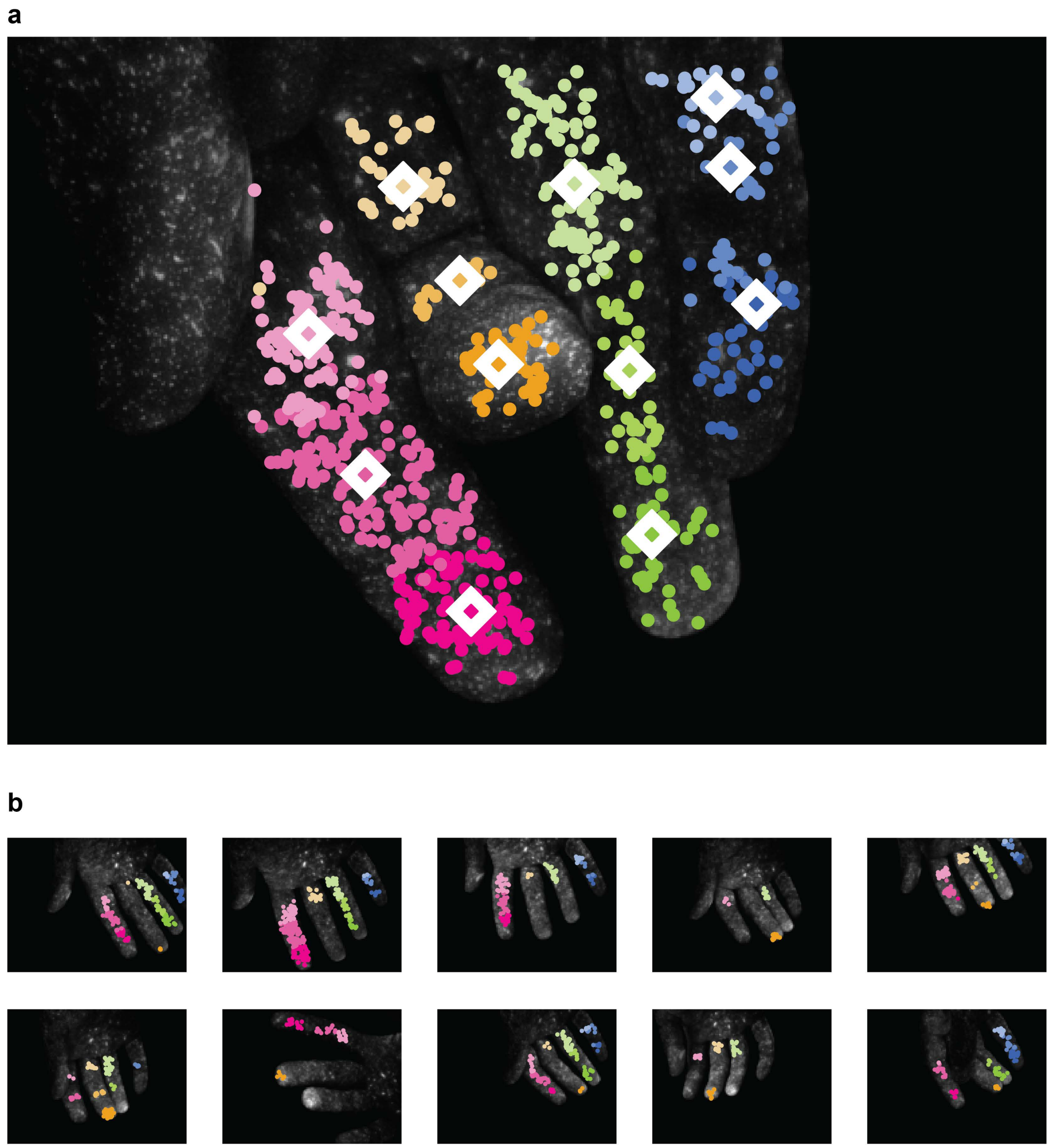

Supp. Fig. 8. Using multiple template images provides more complete coverage. a) A target image with scale-invariant feature transform (SIFT) features (circles) and computed landmark labels (diamonds). Only SIFT features that matched at least one template image in **b** are shown, and color represents the feature's neighborhood membership in the highest-ranked template that it matched. **b)** The 10 template images from which the above target image was labeled. Circles represent matched SIFT features, colored by neighborhood membership. Note that no single template provides coverage of all 12 neighborhoods. Jointly, however, they provide complete coverage.

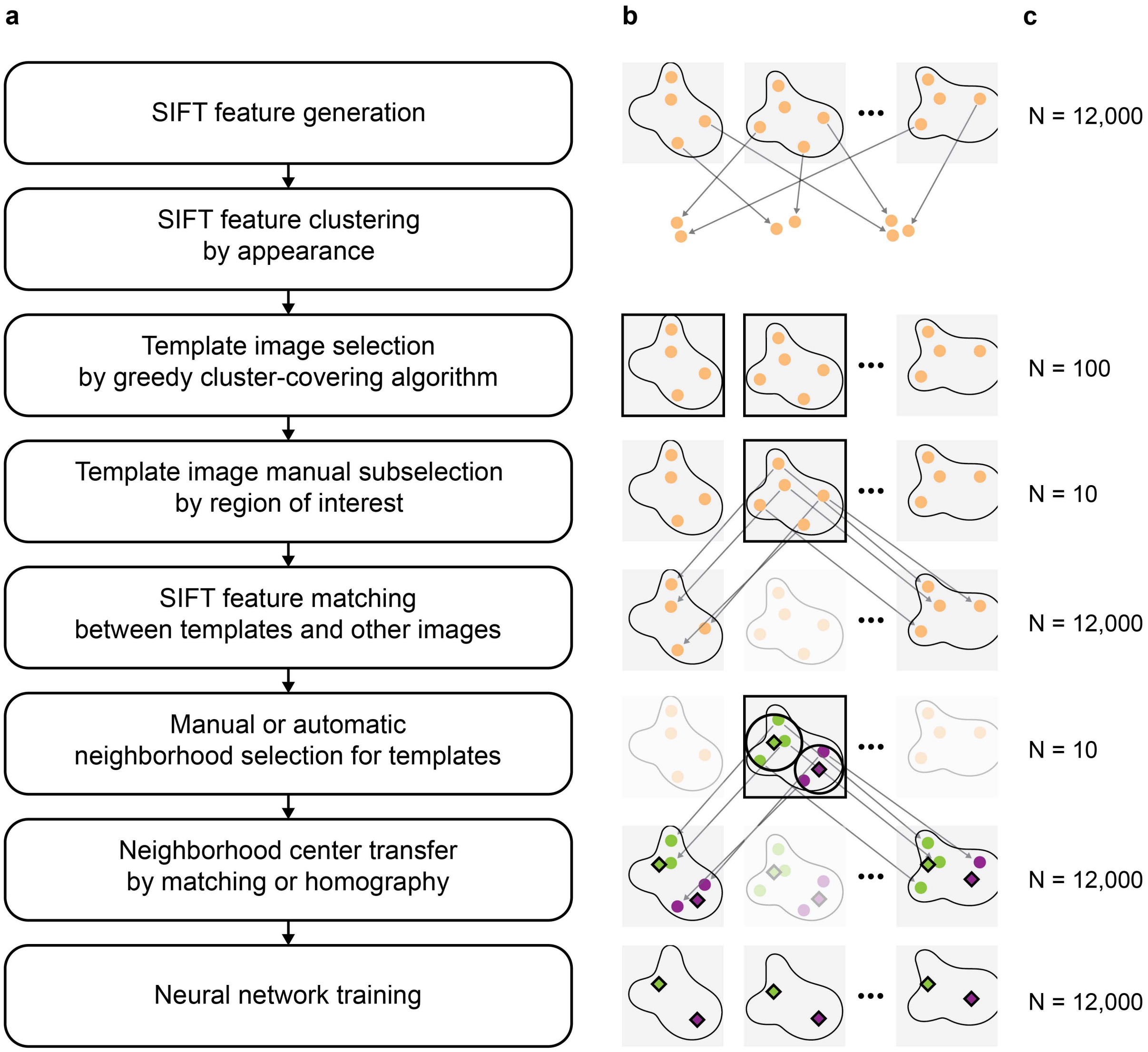

Supp. Fig. 9. Workflow diagram for the parallel labeling approach. a) A flowchart showing the main stages of the computational workflow. **b)** Corresponding graphical representations of each stage. Irregular outline represents an object imaged from different angles. Orange disks represent scale-invariant feature transform (SIFT) features. Gray squares represent images. Bold-outlined gray squares represent images selected as templates. Circular outlines represent neighborhoods selected manually or automatically (radius around a given feature). Bold-outlined diamonds represent neighborhood centers (centroid for manually-selected neighborhoods, and active feature for automatically-selected neighborhoods). **c)** Number of images active in the given stage for our experiments. There were 12,000 images in the entire data set, 100 images selected algorithmically as templates, 10 (out of 100) templates selected manually as regions of interest, 12,000 images matched to the templates, and 12,000 images labeled via matching and used for training.

Training set	Scale optimization	AUC	OKS-mAP	
250 uniform	none	0.24	0.49	
500 uniform	none	0.27	0.52	
1k uniform	none	0.33	0.57	Diverse test set
1k diverse	none	0.51	0.70	
300k diverse	none	**0.89**	**0.95**	
1k diverse	none	0.21	0.34	
380k diverse	none	0.52	0.60	Challenge test set
380k diverse	image-level	0.69	0.75	
380k diverse	clip-level	**0.76**	**0.82**	

Supp. Table 1. Neural network evaluation with the OKS-mAP metric yields similar results as AUC. The object keypoint similarity mean average precision (OKS-mAP; last column) is another standard performance metric developed for human keypoint evaluation. The metric ranges from 0 to 1, with 1 representing the best possible performance. Each row represents a trained neural network model. The top section of the table represents results on the diverse test set. The area under the curve (AUC) values are reproduced from **Fig. 3**. The bottom section represents results on the challenge test set. The AUC values are reproduced from **Fig. 4**. The OKS-mAP metric yields similar results as the AUC metric, supporting the conclusions of the main figures. Source data are provided as a Source Data file.
