# Supplementary material for: Large-scale capture of hidden fluorescent labels for training generalizable markerless motion capture models
Source: Nat Commun. 2023 Sep 26;14:5866. doi: 10.1038/s41467-023-41565-3 (PMC10522643; doi:10.1038/s41467-023-41565-3)
Supplement: Supplementary file 3 — Description of Additional Supplementary Files Document [file 41467_2023_41565_MOESM3_ESM.pdf]

Description of Additional Supplementary Files Document

Supplementary Movie 1. Screencast of video capture software with visible light and UV frames displayed side-by-side. Real-time image capture software while recording a mouse with hidden fluorescent dye on its left hand. The left and right image feeds show the visible and UV illumination frames respectively (i.e., each feed shows only alternate frames). The bottom panel shows information about the video clips that have been captured so far. The far-left panel controls the timing of the different phases of the imaging and illumination cycle.

Supplementary Movie 2. Video of capture rig in operation, slowed down 10x to reveal active illumination source cycling among LED clusters. The capture rig during video recording. Video (1/10th speed) shows how the active illumination source cycles among the nine lighting clusters. All UV sources on the lower five lighting clusters are active simultaneously (they appear out-of-sync due to rolling shutter).

Supplementary Movie 3. Deep learning model trained on hidden fluorescent labels tracks the hand of an unlabeled mouse. Output predictions from a deep learning model trained to detect the left hand of the mouse. The number shown near each landmark is the confidence score. The reaching behavior was seen during training, but the lighting and camera setup are novel.

Supplementary Movie 4. Training data generated using massively parallel visual barcoding. Training data generated using massively parallel labeling with visual barcodes and the manual neighborhood selection pipeline. Both UV and visible images were captured, but for clarity only the visible images are shown. Each dot represents a scale-invariant feature transform (SIFT) match between the given video frame and a template image; dot color encodes which finger segment the match corresponds to in the template. Each diamond represents the centroid of all matches corresponding to a particular finger segment. The images along the bottom are the 10 template images.

Supplementary Movie 5. Deep learning model trained with fluorescent barcoding and manual neighborhood selection tracks the segments of human fingers. Output predictions from a deep learning model trained to detect the finger segments on an unlabeled human hand using the manual neighborhood selection pipeline (training data shown in Supp. Movie 4). Each dot is a landmark predicted by the model. Model was trained by propagating labels from 10 manually-labeled template images to 12,276 training images using fluorescent barcodes. Occluded finger segments do not receive a label.

Supplementary Movie 6. Deep learning model trained with fluorescent barcoding and automatic neighborhood selection tracks 50 unique landmarks. Output predictions from a deep learning model trained to detect 50 landmarks on first digit of an unlabeled human hand using the automatic neighborhood selection pipeline. Each dot is a landmark predicted by the model. Model was trained by propagating labels for 50 automatically selected landmarks in a template image to 12,276 training images using fluorescent barcodes.
