# Supplementary material for: Large-scale capture of hidden fluorescent labels for training generalizable markerless motion capture models
Source: Nat Commun. 2023 Sep 26;14:5866. doi: 10.1038/s41467-023-41565-3 (PMC10522643; doi:10.1038/s41467-023-41565-3)
Supplement: Supplementary file 10 — Reporting Summary [file 41467_2023_41565_MOESM10_ESM.pdf]

nature research

Corresponding author(s): Eiman Azim

Last updated by author(s): May 10, 2023

Reporting Summary

Nature Research wishes to improve the reproducibility of the work that we publish. This form provides structure for consistency and transparency in reporting. For further information on Nature Research policies, see our Editorial Policies and the Editorial Policy Checklist.

Statistics

For all statistical analyses, confirm that the following items are present in the figure legend, table legend, main text, or Methods section.

n/a	Confirmed	
☐	☒	The exact sample size (*n*) for each experimental group/condition, given as a discrete number and unit of measurement
☒	☐	A statement on whether measurements were taken from distinct samples or whether the same sample was measured repeatedly
☒	☐	The statistical test(s) used AND whether they are one- or two-sided<br>*Only common tests should be described solely by name; describe more complex techniques in the Methods section.*
☒	☐	A description of all covariates tested
☒	☐	A description of any assumptions or corrections, such as tests of normality and adjustment for multiple comparisons
☐	☒	A full description of the statistical parameters including central tendency (e.g. means) or other basic estimates (e.g. regression coefficient) AND variation (e.g. standard deviation) or associated estimates of uncertainty (e.g. confidence intervals)
☒	☐	For null hypothesis testing, the test statistic (e.g. *F*, *t*, *r*) with confidence intervals, effect sizes, degrees of freedom and *P* value noted<br>*Give P values as exact values whenever suitable.*
☒	☐	For Bayesian analysis, information on the choice of priors and Markov chain Monte Carlo settings
☒	☐	For hierarchical and complex designs, identification of the appropriate level for tests and full reporting of outcomes
☒	☐	Estimates of effect sizes (e.g. Cohen's *d*, Pearson's *r*), indicating how they were calculated

Our web collection on statistics for biologists contains articles on many of the points above.

Software and code

Policy information about availability of computer code

Data collection	Custom software (https://github.com/azimlabsalk/glowtrack, commit 5404408), Basler Pylon 5 video camera SDK
Data analysis	Custom software (https://github.com/azimlabsalk/glowtrack, commit 5404408), Deepercut landmark detector python package (https://github.com/eldar/pose-tensorflow, commit 6a8f5ee), COCO Analyze landmark detection evaluation python package (https://github.com/matteorr/coco-analyze, commit 9eb8a0a), and Label Studio (https://github.com/heartexlabs/label-studio, v1.5.0)

For manuscripts utilizing custom algorithms or software that are central to the research but not yet described in published literature, software must be made available to editors and reviewers. We strongly encourage code deposition in a community repository (e.g. GitHub). See the Nature Research guidelines for submitting code & software for further information.

Data

Policy information about availability of data

All manuscripts must include a data availability statement. This statement should provide the following information, where applicable:

- Accession codes, unique identifiers, or web links for publicly available datasets
- A list of figures that have associated raw data
- A description of any restrictions on data availability

The main image datasets that support the findings of this study are publicly available online (https://cnl.salk.edu/~dbutler/glowtrack).

Field-specific reporting

Please select the one below that is the best fit for your research. If you are not sure, read the appropriate sections before making your selection.

☒ Life sciences ☐ Behavioural & social sciences ☐ Ecological, evolutionary & environmental sciences

For a reference copy of the document with all sections, see nature.com/documents/nr-reporting-summary-flat.pdf

Life sciences study design

All studies must disclose on these points even when the disclosure is negative.

Sample size	All sample sizes are commensurate with those appearing in the literature for machine learning-based landmark detection.
Data exclusions	Video clips wherein the landmark of interest (mouse hand) was not clearly visible for more than half of the video frames were excluded from the "challenge" data set.
Replication	The code has been packaged with a Dockerfile that reproduces the original software environment. Combined with our open-source custom code and data, the experimental results can be replicated.
Randomization	The division of our data into training and testing subsets was performed using the standard Python pseudo-random number generators.
Blinding	Blinding, in the sense of concealing the experimental conditions under which several groups of data were collected, is not applied here because the standard method for evaluating machine learning algorithms is to test on held out data (see Randomization above).

Reporting for specific materials, systems and methods

We require information from authors about some types of materials, experimental systems and methods used in many studies. Here, indicate whether each material, system or method listed is relevant to your study. If you are not sure if a list item applies to your research, read the appropriate section before selecting a response.

Materials & experimental systems

n/a	Involved in the study	
☒	☐	Antibodies
☒	☐	Eukaryotic cell lines
☒	☐	Palaeontology and archaeology
☐	☒	Animals and other organisms
☐	☒	Human research participants
☒	☐	Clinical data
☒	☐	Dual use research of concern

Methods

n/a	Involved in the study	
☒	☐	ChIP-seq
☒	☐	Flow cytometry
☒	☐	MRI-based neuroimaging

Animals and other organisms

Policy information about studies involving animals; ARRIVE guidelines recommended for reporting animal research

Laboratory animals	Data were obtained from adult C57BL/6 male and female mice (8-12 weeks old) housed on a 12:12 hour light cycle at 65-75°F with 40-60% humidity. Training data and uniform and diverse held out data were collected from 2 male and 2 female mice. Challenge data consisted of videos of 14 male and female mice (exact sex and age distribution is unknown as these videos were randomly selected from archival videos across labs).
Wild animals	No wild animals were used in the study.
Field-collected samples	No field-collected samples were used in the study.
Ethics oversight	Procedures performed in this study were conducted according to US National Institutes of Health guidelines for animal research and were approved by the Institutional Animal Care and Use Committee of The Salk Institute for Biological Studies.

Note that full information on the approval of the study protocol must also be provided in the manuscript.

Human research participants

Policy information about studies involving human research participants

Population characteristics	One male, 33 years old. As only one participant was used as proof-of-concept for our method, disaggregation by sex and/or gender was not relevant.
Recruitment	One human volunteer took part in the study. No compensation was involved. We do not anticipate a self-selection bias with respect to the hand-tracking approach described in the paper.
Ethics oversight	Human work complied with all relevant ethical regulations, was performed with informed consent, and was designated to meet the criteria for Exemption 45 CFR 26.104(d)3 – Benign Behavioral Interventions by the Salk Institute IRB.

Note that full information on the approval of the study protocol must also be provided in the manuscript.
